# The impact of tobacco cessation on buccal mucosa cancer risk: A multi‐centre case–control study in India

**DOI:** 10.1002/ijc.70372

**Published:** 2026-02-09

**Authors:** Gayathri B. Pullat, Bastian Bohrmann, Grace Sarah George, Shubham Dikshit, Arjun Singh, Pankaj Chaturvedi, Rajesh Dikshit, Sarah Lewington, Sharayu Mhatre

**Affiliations:** ^1^ Division of Molecular Epidemiology and Population Genomics, Centre for Cancer Epidemiology Tata Memorial Centre Navi Mumbai India; ^2^ Department of Health Sciences Homi Bhabha National Institute (HBNI) Mumbai India; ^3^ Clinical Trial Service Unit and Epidemiological Studies Unit (CTSU), Nuffield Department of Population Health University of Oxford Oxford UK; ^4^ Health Data Research UK Oxford University of Oxford Oxford UK; ^5^ Unit for Strengthening Cause of Death Data, Centre for Cancer Epidemiology Tata Memorial Centre Navi Mumbai India; ^6^ Department for Head and Neck Oncology Tata Memorial Centre Mumbai India; ^7^ Advanced Centre for Treatment, Research and Education in Cancer Tata Memorial Centre Navi Mumbai India; ^8^ Centre for Cancer Epidemiology Tata Memorial Centre Navi Mumbai India

**Keywords:** abstinence, chewing tobacco, observational study, oral cancer, quitting

## Abstract

While the association between tobacco consumption and oral cancer is well established, the effect of tobacco cessation, particularly chewing tobacco, is less extensively studied. We aim to explore the effect of tobacco cessation on the risk of buccal mucosa cancer (BMC). A case–control study was conducted across five cancer centres in India. We enrolled 2320 BMC and 2302 frequency‐matched controls. Information was collected on smoking and chewing (products, duration and quitting). Unconditional logistic regression models were used to estimate odds ratios (OR) and 95% confidence intervals (95% CI) of the effect of tobacco cessation compared to current users, after adjusting for potential confounders. For both smoking and chewing, the odds reduced rapidly after 5 years of quitting, so that those who had quit smoking ≥10 years ago had only 0.39 (95% CI 0.28–0.54) the odds of BMC compared to those who continued to smoke, and those who quit chewing ≥10 years ago had 0.58 (95% CI 0.43–0.81) the odds of BMC compared to those who continued to chew. Chewing with areca nut was associated with almost double the risk of chewing without (OR 1.86, 95% CI 1.53–2.26) but the rate of reduction of risk with quitting was similar. These findings show clearly the benefits of quitting and inform policies that emphasize chewing tobacco cessation, given its widespread prevalence in India.

## Graphical abstract

What's new?

While the association between tobacco consumption and oral cancer is well established, the effect of cessation, particularly of chewing tobacco, the most common tobacco product in South Asia, is understudied. This case–control study in an Indian population demonstrates that a longer duration of tobacco smoking and chewing cessation is associated with a lower risk of buccal mucosa cancer, although it remains higher than in never‐users. Chewing tobacco with areca nut almost doubled the cancer risk, but it was similarly reduced by quitting. These findings emphasize that while all forms of tobacco are harmful, quitting can lead to partial risk reversal.

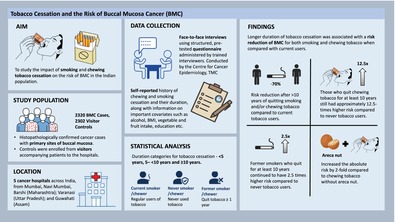

**Abbreviations**
BMC: buccal mucosa cancer
CCE: Centre of Cancer Epidemiology
CI: confidence interval
COTPA: Cigarettes and Other Tobacco Products Act
DMG: Disease Management Groups
IARC: International Agency of Research on Cancer
ICD: International Classification of Diseases
OC: oral cancer
OR: odds ratio
SD: standard deviation
TSNA: tobacco‐specific nitrosamines
WHO: World Health Organization

## 1 INTRODUCTION

Tobacco use in multiple forms is an established cause for many cancer types.^1^ Use of both smoked and smokeless tobacco products (including chewed tobacco) is a leading preventable cause of cancer.^2^, ^3^ Globally there have been continuous efforts to reduce the use of tobacco including the WHO Framework Convention on Tobacco control, a treaty which is signed by 181 countries.^4^ Despite these efforts, tobacco use remains common worldwide.^5^, ^6^ The burden of tobacco is particularly high in countries in the South Asian region with dual use of tobacco products. For example, in Bangladesh, approximately 2%–3% of tobacco users are both smokers and chewing tobacco users, while in India, approximately one‐fifth of males are dual users of tobacco.^6^ The high prevalence of dual use of tobacco in India is reflected by high incidence rates of oral cancer (OC) with the number of OCs estimated to be nearly 150,000 in 2022.^7^ In India, buccal mucosa cancer (BMC) is one of the most prevalent subsites of OC.^8^ The efforts to reduce the burden of oral cavity cancer at present are at primary prevention level through tobacco control and implementation of tobacco cessation programmes.

As the number of tobacco users is high in India (approximately 30%),^9^ it is important to implement tobacco cessation programmes that are effective. While primary prevention programmes for OC utilize well documented evidence on tobacco use and risk of oral cavity cancer^10^ (and many other cancer types), limited data are available from India on the risks of tobacco chewing on cancer risk for OC (and other cancer sites). Such data will provide evidence for current tobacco chewers to quit chewing tobacco. Similarly, although there is ample evidence on the benefits of quitting (cessation of) smoking tobacco with risk of various cancer types (with risk decreasing with increasing duration of quitting), there is a paucity of evidence on the potential benefits of quitting chewing tobacco that needs to be considered for inclusion in these prevention programmes.^11^ Furthermore, since BMC is predominantly attributed to tobacco chewing, we specifically examined the risks of tobacco chewing and benefits of quitting on BMC risk. To our knowledge, no studies have addressed tobacco cessation specific to this subsite.

Therefore, we investigated the association of smoking and chewing tobacco and of duration of quitting with the risk of BMC in an Indian population.

## 2 METHODS

### 2.1 Study design and participants

For this hospital‐based case control study, the enrolment was carried out in five cancer centres, situated in the cities of Mumbai, Navi Mumbai, Barshi, Varanasi and Guwahati in India, during the time period 2010–2022 (Table S1). Both men and women who were residents of India for at least 1 year, and aged 19–75 years, were enrolled. Cases with primary sites of buccal mucosa (ICD‐O: C06.0‐C06.2, and C03.0‐C03.1) were histopathologically confirmed, and were prospectively enrolled along with controls adhering to the checklist that ensures their eligibility (Table S2). For cases, the date of diagnosis was less than or equal to 6 months from the date of enrolment and have been residents of India for a year. Controls were recruited from all Disease Management Groups (DMGs)—specialized units managing specific cancer sites—within the cancer centres. Any visitors accompanying the patient from the respective study centres, which enrolled the cases, were included as a control. The visitor controls belonged to the group of friends, neighbours, second‐degree relatives of the patients of other cancer sites. (Table S2). To maintain balance in order to select the controls from the study base, no single DMG contributed more than 30% of the total control population. The controls were frequency‐matched to the cases on age (±10 years age interval) and region of current residence (South, Central, Northeast, North and West). Graphical representation of these regions can be found in Figure S1.

### 2.2 Procedures

Information regarding the tobacco exposure as well as on the covariates were collected through a structured, pre‐tested questionnaire, administered by trained interviewers.^12^, ^13^ To ensure consistency in data collection, interviewers were given training periodically, using a standardized in‐house manual. Four percent of participants were re‐interviewed to ensure data reproducibility. The kappa statistics percentage agreement was 98% for smoking and 94% for chewing tobacco, indicating strong agreement. Data from the questionnaire, along with any missing data, was cross‐checked by the interviewer, project coordinator as well as the data manager. Double data entry was performed to ensure the accuracy of data entry.

### 2.3 History of tobacco consumption

Details of 11 smoking products, namely—cigarette, bidi, cheroot, cigar, roll your own, chutta, reverse chutta, dhuranti, reverse dhuranti, hookah and hookli/chillum—were collected for the age of participants when they started using the product, age when they stopped, number per day and how many days in a week they consumed it. Similarly, for smokeless/chewing tobacco products, usage information on a plethora of region‐specific tobacco products was collected, such as the age at initiation, age at cessation, the quantity consumed (number per day), and the duration of consumption (in a week). The chewing tobacco products were subsequently grouped chewing tobacco with and without areca nut. Tobacco products with areca nut included—betel quid, gutkha, kharra, manipuri, dohra, mawa; and those without areca nut included—zarda, khiwam and paan masala (Table S3).

Information on tobacco consumption was collected for various types of region‐specific tobacco products, both smoking and chewing, which was further classified based on the presence/absence of areca nut. Ever users of either chewing or smoking tobacco were individuals who have consumed tobacco currently or previously at least once a week for at least a span of 6 months. Never users were those that had never consumed any tobacco, or consumed it only for <1 time per week during their lifetime.

Current smokers and chewers were participants who reported regularly using tobacco at the time of recruitment or had quit up to 12 months before recruitment. Former users were defined as participants who had quit use of tobacco at least 1 year prior to recruitment; former users were subsequently categorized into multiple groups according to their duration since quitting.

Additional details on exposure assessment have been mentioned in the section on statistical analysis.

### 2.4 Statistical analysis

Analyses included 2320 BMC cases and 2302 visitor controls. All models were adjusted for age (continuous), gender, self‐reported education level (<5 years, ≥5 years), maximum duration of smoking tobacco use (continuous, where chewing tobacco was the exposure) and maximum duration of chewing tobacco use (continuous, where smoking tobacco was the exposure), BMI (categorical—normal [18.5–24.9 kg/m^2^], severely underweight [<16.5 kg/m^2^], underweight [16.5–18.5 kg/m^2^], overweight [25–30.0 kg/m^2^], obese [>30.0 kg/m^2^]), fruit and vegetable intake (<2 versus ≥2 portions per day), and alcohol consumption (grams per day). For further details on covariate definition and categorization, see Extended methods, Appendix S2.

The cessation duration among the former users was categorized into three groups: those who quit <5 years, 5–<10 years and ≥10 years ago (from the date of interview). Subsequently, participants who had quit tobacco for at least 1 year at the time of interview were categorized as former users. Details on the calculation of cessation duration have been explained in Appendix S1.

Multivariate unconditional logistic regression was used to derive adjusted odds ratios (OR) and 95% confidence intervals (CI).

Where there are five categories of tobacco usage, logistic regression arbitrarily designates one the reference group (current users in our analyses) and yields the variances and co‐variances of the log OR values for the four others (never users and three groups of former users). From these variances and co‐variances, the *variance of the log risk* in each of the groups (one being the reference group) was estimated.^14^, ^15^ These five variances do not depend on which group was designated the reference group, and each is approximately the inverse of the number of cases in its group (Table S5A–C). If the variance of the log risk in one of these five groups is V, the group‐specific 95% CI for that group's OR runs from OR_exp_ (−1·96√V) to OR_exp_ (1·96√V). For the reference group (with OR = 1), as for all other groups, its group‐specific CI describes how chance could have affected the log risk in that one group.

The risk per 10 and 20 years of cessation of both smoking and chewing tobacco for BMC was also estimated using the continuous, non‐categorized cessation duration variable of smoking tobacco and chewing tobacco.

Due to a low number of cases in the categories of former users for several of the tobacco products, analyses compared BMC risk among never to ever users of the individual tobacco products (Table S6).

To explore the impact of quitting one type of tobacco in dual users, we carried out analyses for risk of BMC with levels of smoking cessation duration in those that continued to chew, as well as of chewing cessation duration in those that continued to smoke (Table S7A,B). The reference category for both analyses was current dual users of smoking and chewing tobacco.

All analyses were performed using STATA®15.0.^16^ The plots were illustrated using the package ‘Jasper’ in RStudio.^17^ The map illustration was designed using iipmaps.^18^

## 3 RESULTS

Baseline characteristics of participants are summarized in Table 1. This study included 2320 cases and 2302 controls, the majority of which were men (88%). Mean (standard deviation [SD]) age was 46.4 years (9.9) for cases and 43.3 years (10.5) for controls (*p* < .001).

**TABLE 1:** Distribution of the study population based on socio‐demographic variables.

		BMC cases (%) (N_1_ = 2320)	Controls (%) (N_2_ = 2302)	*p*‐value
Age (in years)	<30	107 (4.61)	262 (11.38)	
	30–40	601 (25.91)	712 (30.93)	
	40–50	837 (36.08)	767 (33.32)	<.001
	50–60	566 (24.40)	420 (18.25)	
	> = 60	209 (9.01)	141 (6.13)	
	Mean (±SD)	46.4 (±9.86)	43.3 (±10.45)	<.001
Gender	Males	2039 (87.89)	2037 (88.49)	
	Females	281 (12.11)	265 (11.51)	.527
Education^a^	≥5 years of education	1669 (71.94)	2085 (90.57)	
	<5 years of education	651 (28.06)	217 (9.43)	<.001
Region of residence at the time of enrolment^b^	West	878 (37.84)	949 (41.23)	
	North	821 (35.39)	555 (24.11)	
	North‐East	460 (19.83)	647 (28.11)	
	Central	141 (6.08)	124 (5.39)	<.001
	South	16 (0.69)	21 (0.91)	
	Non‐residents of India	3 (0.13)	3 (0.13)	
	Missing	1 (0.04)	3 (0.13)	
BMI (kg/m^2^)	Normal (18.5–24.9)	1284 (55.34)	1081 (46.96)	
	Obese (>30.0)	100 (4.31)	213 (9.25)	
	Overweight (24.9–30.0)	488 (21.03)	818 (35.53)	
	Underweight (16.5–18.5)	227 (9.78)	112 (4.87)	<.001
	Severely underweight (<16.5)	133 (5.73)	18 (0.78)	
	Missing	88 (3.79)	60 (2.61)	
Any alcohol consumption	Yes	895 (38.58)	519 (22.55)	
	No	1425 (61.42)	1783 (77.45)	<.001
	Grams consumed per day			
	Mean (±SD)	34.59 (±51.79)	28.08 (±49.88)	.021
Any tobacco smoking^c^	Yes	775 (33.41)	582 (25.28)	
	No	1545 (66.59)	1720 (74.72)	<.001
	Smoking duration (in years)			
	Mean (±SD)	16.67 (±11.30)	15.89 (±11.15)	.201
Any tobacco chewing^d^	Yes	2089 (90.04)	792 (34.40)	
	No	231 (9.96)	1510 (65.60)	<.001
	Chewing duration (in years)			
	Mean (±SD)	20.17 (±11.22)	17.30 (±11.07)	<.001
Any fruit intake (portions per day)	≥2	1276 (55.00)	1252 (54.39)	
	<2	874 (37.67)	912 (39.62)	.117
	Missing	170 (7.33)	138 (5.99)	
Any vegetable intake (portion per day)	≥2	1760 (75.86)	1544 (67.07)	
	<2	391 (16.85)	622 (27.02)	<.001
	Missing	169 (7.28)	136 (5.91)	

*Note*: Data are represented as n (%) unless otherwise stated.

^a^ Categories for education has been made by compiling the following: 5–8 years of schooling—have only studied till 5th/6th/7th or 8th grade in school. High school—completed their education till 12th grade or diploma. Illiterate—attained no formal education and does not know to read and write in any language. College graduation or more—has successfully completed graduation and holds a graduate certificate. Less than 5 years schooling—studied anything less than 5th grade. Literate—attained no formal education but knows how to read and write.

^b^ Region of residence at the time of enrolment (Regions and their respective states). West—Goa, Gujarat, Daman & Diu, Dadra and Nagar Haveli, Maharashtra. North—Uttar Pradesh, Bihar, Delhi, Haryana, Himachal Pradesh, Jammu and Kashmir, Punjab, Rajasthan, Chandigarh, and Uttarakhand. North‐East—Arunachal Pradesh, Assam, Meghalaya, Nagaland, Manipur, Tripura, West Bengal, Jharkhand, and Orissa. Central—Madhya Pradesh and Chhattisgarh. South—Andhra Pradesh, Karnataka, Kerala, Lakshadweep, Tamil Nadu, and Telangana. Non‐residents of India—participants whose current residence was not reported as India.

^c^ Any smoking tobacco considers the history of consumption of any tobacco product that is smoked.

^d^ Any chewing tobacco considers the history of consumption of any tobacco product that is chewed.

Overall, 62% reported ever using some chewing tobacco product in their lifetime, with a higher proportion among cases (90%) than controls (34%), and a longer duration (mean 20.2 [SD 11.3] vs. 15.8 [11.2] years; *p* < .001) (Table 1). Among ever tobacco chewers, quitting was more common in cases (29%) than in controls (23%). The most commonly used chewing product was tobacco with lime (61%), followed by gutkha (39%), and betel quid with tobacco (32%), mawa (6%) and mishri (4%). Among these, tobacco with lime had the lowest quitting rates among overall users (25%) and among cases (26%) and controls (20%) (Table S4).

Overall, nearly one‐third (29%) of the total study population reported smoking tobacco, with a notably higher proportion of cases (33%) than controls (25%). Mean (SD) tobacco smoking duration was similar for cases (16.7 years [11.3]) and controls (15.9 years [11.2], *p* = .20). For former smokers, the overall quitting rate was higher in controls (41%) compared to cases (38%). Among all smoking tobacco products, cigarettes were the most used product (75%), followed by bidi (40%). Cigarettes had a high quitting rate (40% of cases and 42% of controls among the cigarette users), followed by bidi (34% of cases and 38% of controls among the bidi users) (Table S4).

The quitting rate among chewers was 27%, much lower than that of smokers (39%). Products like gutkha and betel quid with tobacco had relatively high usage, but quitting rates among them remained low—39% and 34%, respectively. However, cigarette users, who formed 22% of the population, had a moderately high quitting rate of 41%. Prevalence of quitters and users of tobacco products in our study can be found in Appendix S3.

A higher proportion of cases (28%) had less than 5 years of education, compared with controls (9%; *p* < .001). Most participants were from West India (38% of cases, 41% of controls) or North India (36% of cases, 24% of controls). The proportion of participants with a normal BMI (i.e., ≥18.5 and <25 kg/m^2^) was higher among cases (55%) compared to controls (47%). By contrast, underweight (BMI ≥16.5 and <18.5 kg/m^2^) and severe underweight (BMI <16.5 kg/m^2^) were more common among cases (10% and 6%, respectively) than controls (5% and 1%, respectively) (Table 1).

Alcohol drinking was higher among cases (39%) than controls (23%) and among those who did drink, the consumption was higher in cases (35 vs. 28 g/day; *p* = .02). Controls had, on average, lower vegetable intake—only 67% of controls consumed vegetables at a frequency of less than twice per day, compared to 76% of cases (*p* < .001)—but had similar fruit intake (38% vs. 40%; *p* = .12) (Table 1).

**FIGURE 1:**
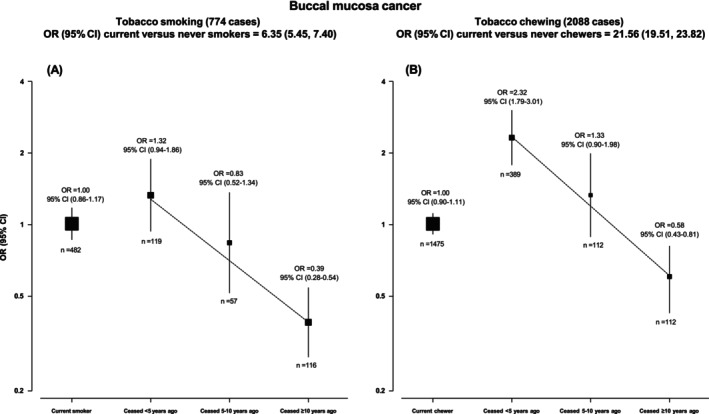
(A) Cessation of smoking tobacco and the risk of BMC, when compared with current smokers. Adjusted for age, gender, education, BMI, vegetable intake in frequency per day, fruit intake in frequency per day, grams of alcohol consumed per day, maximum duration of chewing tobacco consumption. Error bar represents group‐specific floating confidence intervals. Numbers below and above the square indicate cases in that category and their odds ratio, respectively. Counts include dual users and quitters; they are not limited to exclusive smoking tobacco users. (B) Cessation of chewing tobacco and the risk of BMC, when compared with current chewers. Adjusted for age, gender, education, BMI, vegetable intake in frequency per day, fruit intake in frequency per day, grams of alcohol consumed per day, maximum duration of smoking tobacco consumption. Error bar represents group‐specific floating confidence intervals. Numbers below and above the square indicate cases in that category and their odds ratio respectively. Counts include dual users and quitters; they are not limited to exclusive chewing tobacco users.

Figure 1 illustrates the reduction in OR of BMC at different smoking (Figure 1A), and chewing durations of quitting (Figure 1B), relative to current tobacco users. While current smokers had 6.35 (95% CI 5.45–7.40) times the odds of BMC compared to never smokers, quitting substantially reduced the odds, almost log‐linearly, so that those who had quit smoking 5–<10 years ago had 0.83 (0.52–1.34) and ≥10 years ago had 0.39 (0.28–0.54) the odds of current smokers. The OR (95% CI) of BMC among current chewers compared to never chewers was 21.56 (19.51–23.82). The odds of BMC were lower with longer time since cessation (OR [95% CI] of 2.32 [1.79–3.01] for those who quit <5 years ago and 0.58 [0.43–0.81], both compared to current chewers) (Figure 1, Table S5A,B).

Despite such large reductions in odds of BMC with quitting, especially after longer periods of cessation, the odds for those who had quit smoking and chewing tobacco for over 10 years were still 2.5 and 12.5 times higher, respectively, compared to the respective never users, as shown in Figure 1.

Longer duration of quitting was associated with a lower risk of BMC for both smoking and chewing so that the OR (95% CI) per 10 years of quitting compared with current smokers was 0.68 (0.56–0.81) for smoking and 0.77 (0.65–0.91) for chewing, and OR (95% CI) per 20 years of quitting was 0.46 (0.32–0.66) for smoking and 0.59 (0.42–0.83) for chewing.

**FIGURE 2:**
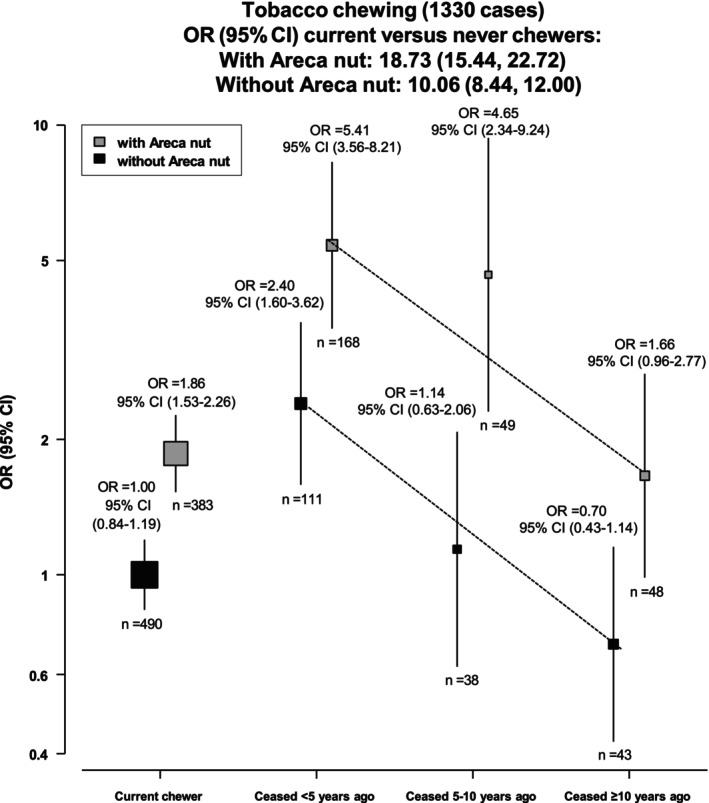
Cessation of chewing tobacco with and without areca and the risk of BMC, when compared with current chewers without areca nut. Adjusted for age, gender, education, BMI, vegetable intake in frequency per day, fruit intake in frequency per day, grams of alcohol consumed per day, maximum duration of chewing tobacco and smoking tobacco consumption. Error bars represent group‐specific floating confidence intervals. Numbers below the square indicate cases in that category and the numbers above indicate their respective odds ratios. BMC, buccal mucosa cancer. Counts contain dual users of chewing and smoking tobacco.

Figure 2 shows the association of tobacco chewing status with risk of BMC, stratified by areca nut consumption. Longer duration of chewing tobacco cessation was inversely associated with BMC risk, regardless of areca nut co‐consumption, though participants who also had consumed areca nut were at a higher absolute risk than those who had chewed tobacco without areca nut (indicated by higher ORs for ever‐chewers with areca nut, and at every given level of chewing duration). Compared to current chewers without areca nut, recent quitters (<5 years ago) who had chewed tobacco with areca nut had a higher risk of BMC (OR [95% CI]: 5.41 [3.56–8.21]) than recent quitters who chewed without areca nut (2.40 [1.60–3.62]). After ≥10 years of cessation, ORs (95% CIs) were 1.66 (0.96–2.77) with areca nut and 0.70 (0.43–1.14) without areca nut, which suggests a similar reduction in log OR for quitters who had chewed tobacco with and without areca nut of approximately 70%. (Table S5C)

All products showed a lower risk among never‐users compared with the respective ever‐users (Table S6). The odds of BMC among dual users, who had quit either chewing or smoking for at least 10 years of quitting but carried on consuming tobacco in the other form, yielded similar associations to the main analyses (Figure 1A, Appendix S7A,B).

## 4 DISCUSSION

This study supports evidence suggesting excess risk of BMC associated with both smoking and particularly chewing tobacco, and is to our knowledge the first to demonstrate that longer duration of cessation of tobacco smoking and chewing is significantly associated with lower risk of BMC, compared to the respective current users. Our findings suggest that after at least 10 years of cessation, the risk of BMC is reduced by approximately half compared to current or recent tobacco users. This was regardless of whether or not (previous) chewing tobacco users also consumed areca nut, though the addition of areca nut was associated with a higher relative risk in current chewers and at all durations of cessation.

However, these results emphasize the still substantially higher risk of BMC in current and former smoking and chewing tobacco users compared to never smokers and chewers. For example, those who quit chewing tobacco for at least 10 years still had approximately 12.5‐times higher risk compared to never chewers. These findings, therefore, highlight the importance of tobacco cessation in the mitigation of BMC, and simultaneously provide strong evidence for the detrimental health effects of tobacco initiation, which can only partly be mitigated by cessation.

The IARC Handbook on OC prevention and cancer prevention have summarized a number of studies that evaluated the impact of quitting tobacco smoking on the risk of developing OC.^11^, ^19^ Since IARC handbook volume 11, two additional cohort studies^20^, ^21^ and a pooled analysis of 17 case–control studies^22^ have been identified in volume 19 for smoking cessation, which showed a reduction in risk with increasing time since quitting. Additionally, a meta‐analysis which explored the association of smoking cessation with the risk of head and neck cancers reported a summary RR (95% CI) of 0.40 (0.35–0.46) among former smokers when compared to current smokers, with the risk being reduced by half for each 10 years of smoking cessation.^23^ However, these findings were mainly based upon cessation of cigarette smoking and did not study other smoking products, which are common in India.

In contrast to the availability of sufficient literature on cessation of tobacco smoking, only a few research articles are available for cessation of tobacco chewing.^24^, ^25^, ^26^, ^27^ These studies were underpowered and only considered betel quid with tobacco for chewing tobacco. Interestingly, these studies, like ours, have reported a higher risk during the early years post‐cessation compared to current users, likely due to reverse causality. In the present study, recent quitters of tobacco chewing had a twofold higher BMC risk compared to current chewers, but the strong inverse association with duration of quitting beyond 5 years is evidence of reverse causality, whereby individuals may have stopped using tobacco following the onset of pre‐malignant or malignant disease (rather than developing BMC *because* they quit).^24^, ^25^

The levels of nicotine and tobacco‐specific N‐nitrosamines (TSNA) in chewing tobacco products are extremely high on average, which enhances their addictiveness and carcinogenicity. Total nicotine content across chewing products ranges from 0.45 to 35.1 mg/g, while total carcinogenic TSNA levels range from 0.09 to 102 μg/g.^28^ Among the TSNAs, evidence suggests that *N*′‐nitrosonornicotine or NNN may play an important role as a cause of cancer in chewing tobacco products.^2^ After the hydroxylation of NNN by CYP2A6 and CYP2A13 enzymes at the pyrrolidine ring, 5′‐hydroxyNNN—a highly reactive intermediate—is formed. This undergoes immediate ring opening, which leads to the formation of an electrophilic diazohydroxide, which reacts with DNA, forming DNA adducts. These adducts, at the end, interfere with DNA replication, causing mutations which would eventually facilitate the development of tumours.^2^ The levels of NNN are comparatively high among chewing tobacco products in India, with the concentration ranging from 0.06 to 76 μg/g.^28^ This, coupled with the varied mode of intake, and other additions such as alkaline modifiers such as slaked lime and areca nut, may contribute to the increased risk of developing OC.

The chewing of areca nut alongside that of tobacco was associated with about twofold higher odds and this difference persisted even after long‐term cessation, suggesting the major carcinogenic effect of areca nut. Areca nut is classified as a group 1 carcinogen and is an established cause of OC.^29^ It is usually placed inside the mouth, and is sucked and chewed slowly, alone or along with a combination of additives such as tobacco, catechu, betel leaf and slaked lime (Table S3). This leads to the repeated exposure of the mouth lining to the chemicals from the areca nut preparation. The coarse texture of the nut causes micro tears in the oral mucosa, making it easier for the harmful substances to go deeper into the tissue, triggering inflammation. Continued chewing can worsen the inflammation at the placement site, which can lead to OC.^30^

Efforts to regulate and control tobacco should focus not only on smoking but also on chewed products. These are not only used more commonly in certain populations, but also have a fewer proportion of users attempting to quit. To aid the quitting process, there are a range of in‐person and digital/telephonic interventions available in India. These include tobacco cessation clinics and regional tobacco quit line services serving almost all states and union territories of India.^31^, ^32^ While growing steadily, these services need more penetration and strengthening, along with delivery of tailored information to result in improved outcomes. The ‘mCessation’ programme, a text message‐based mobile health initiative implemented by the ministry of health and family welfare to help tobacco users to quit, reported a quitting rate of about 20% by the users within 4–6 months registration.^33^ However, while excise duties on tobacco products have risen by 11%–72% over the past decade,^34^ tax increases on bidis and chewing tobacco products remain disproportionately low, underscoring the need for a more comprehensive approach to tobacco control. Our results provide a first of its kind evidence that demonstrates reduction in long term cancer risk with smokeless tobacco cessation, and should inform ongoing strategies. Additionally, these should be continuous, region‐specific, and tailored to address the diverse range of region‐specific tobacco products used across India.

Over the past two decades, India's chewing tobacco industry has grown exponentially, including both the unorganized and organized sectors. To regulate this, a comprehensive Cigarettes and Other Tobacco Products Act 26 or COTPA was introduced a few decades ago.^35^ At the time, several provisions of the COTPA were primarily based on evidence from smoked tobacco from Western countries. Key mandates of COTPA, such as labelling nicotine and tar content, affixing excise stamps, and requiring manufacturing licenses, are not wholly applied to chewing tobacco products, especially the unorganized products.^36^ This lacuna has resulted in Indian states relying on a combination of other laws like the consumer protection, food safety and environmental laws to regulate tobacco use, manufacture and sale.^37^ Despite this, the unorganized sector continues to operate largely outside the scope of regulatory oversight.^36^ Regular updates of the COTPA and other laws are needed to strengthen its implementation and keep it relevant.

This study has several strengths. First, it includes a large number of cases and controls, as well as a significant proportion of quitters of tobacco, which allows stratification by cessation duration and by co‐consumption of areca nut. Participants were recruited from different regions of India, which allowed the collection of extensive information on different types of smoked and chewed tobacco. All cases were histologically confirmed and controls were sampled from the same underlying population so as to minimize selection bias. However, this study also has some limitations. Despite its large number of participants, we could not evaluate associations with longer periods of quitting in greater detail due to the low number of participants who quit for more than 10 years. This highlights an opportunity for interventions needed to make the quitting sustainable over longer periods of time. Tobacco habits (including age at quitting) were self‐reported, which might induce reporting bias through social desirability, particularly among cases. Due to participants being predominantly male, analyses could not be carried out separately for men and women. Further, we could not account for the chemical constituents, commerciality, or the frequency of intake of these tobacco products, which influence the additive potential of the products and impact cessation outcomes. A key limitation of this study is the possibility of reverse causality, particularly among recent quitters of chewing and smoking tobacco due to the appearance of early symptoms or diagnosis of pre‐malignant or malignant conditions. This could lead to an apparent increase in risk among recent quitters. However, to address this issue, we used multiple categories of duration of quitting (‘<5 years’, 5–<10 years and ‘≥10 years’) in the analysis, which allowed us to distinguish the excess risk among recent quitters due to reverse causality from the long‐term benefits of cessation. Nevertheless, future prospective cohort studies with careful exclusion of cases occurring shortly after cessation will be needed to distinguish reverse causality from true causal effects. Moreover, subgroup analyses could not be pursued for important covariates, such as age categories, sex and other key characteristics, due to a low number of cases and controls in each subgroup. Analysis of dual users shown in Table S7A,B should be interpreted cautiously due to small numbers in each subgroup.

In conclusion, this study presents the potential benefits of quitting both smoked and chewed tobacco, with larger benefits for longer durations of cessation. These findings can be used for motivational counselling efforts, emphasizing that all forms of tobacco are harmful but that quitting can lead to risk reversal. Additionally, our results can support the development of future government guidelines and intervention policies, encouraging them to place greater emphasis on tobacco cessation.

## AUTHOR CONTRIBUTIONS

**Gayathri B. Pullat:** Writing – original draft; methodology; writing – review and editing; formal analysis. **Bastian Bohrmann:** Investigation; writing – original draft; methodology; visualization; writing – review and editing; formal analysis. **Grace Sarah George:** Investigation; writing – original draft; writing – review and editing; formal analysis; methodology. **Shubham Dikshit:** Writing – review and editing. **Arjun Singh:** Writing – original draft; writing – review and editing; formal analysis. **Pankaj Chaturvedi:** Investigation; writing – original draft; writing – review and editing. **Rajesh Dikshit:** Conceptualization; investigation; writing – original draft; funding acquisition; methodology; writing – review and editing; formal analysis; supervision. **Sarah Lewington:** Writing – original draft; writing – review and editing; investigation; validation; formal analysis; supervision. **Sharayu Mhatre:** Conceptualization; investigation; funding acquisition; writing – review and editing; validation; methodology; formal analysis; data curation; supervision.

## FUNDING INFORMATION

This study was funded by the Department of Health Research, Indian Council of Medical Research, New Delhi (grant no. ICMR/EU/13/2012/NCD‐III). The funding source of this research study had no role in the study design, collection, data analysis, data interpretation, writing of the report or submission of the paper in the journal. SL reports grants from: the UK Medical Research Council (MRC) and HDR UK (HDRUK2023.0028) funded by the MRC, Engineering and Physical Sciences Research Council, Economic and Social Research Council, Department of Health and Social Care (England), Chief Scientist Office of the Scottish Government Health and Social Care Directorates, Health and Social Care Research and Development Division (Welsh Government), Public Health Agency (Northern Ireland), British Heart Foundation (BHF) and Cancer Research UK; and research funding from the US Centers for Disease Control and Prevention Foundation (with support from Amgen) and from the World Health Organization during the conduct of the study, all outside the submitted work. The Clinical Trial Service Unit and Epidemiological Studies Unit (CTSU) receives research grants from industry that are governed by University of Oxford contracts that protect its independence and has a staff policy of not taking personal payments from industry; further details can be found at (https://www.ndph.ox.ac.uk/files/about/ndph-independence-of-research-policy-jun-20.pdf). BB is supported by an HDR UK Early Career Fellowship, specifically as part of the HDR UK Molecules to Health Records Driver Programme (HDRUK.2023.0028). Health Data Research UK is funded by UK Research and Innovation, the Medical Research Council, the British Heart Foundation, Cancer Research UK, the National Institute for Health and Care Research, the Economic and Social Research Council, the Engineering and Physical Sciences Research Council, Health and Care Research Wales, Health and Social Care Research and Development Division (Public Health Agency, Northern Ireland), Chief Scientist Office of the Scottish Government Health and Social Care Directorates.

## CONFLICT OF INTEREST STATEMENT

The authors declare no conflict of interest.

## ETHICS STATEMENT

This research work was approved by the respective Institutional Ethics Committees (IEC) of the institutes that participated in the study. The study was conducted in accordance with the Declaration of Helsinki. Written informed consent was obtained from all participants.

## Supporting information


**Data S1.** Supporting Information.

## Data Availability

All source code is publicly available on GitHub (https://github.com/CCE‐Consortium/Tobacco‐Cessation‐And‐Risk‐Of‐Buccal‐Mucosa‐Cancer.git). The other data that support the findings of this study are available from the corresponding author upon reasonable request.
