# Supplementary material for: The impact of tobacco cessation on buccal mucosa cancer risk: A multi‐centre case–control study in India
Source: Int J Cancer. 2026 Feb 9;159(2):368–77. doi: 10.1002/ijc.70372 (PMC13193520; doi:10.1002/ijc.70372)
Supplement: Supplementary file 1 — Data S1. Supporting Information. [file IJC-159-368-s001.pdf]

Appendix Material:

Title: The impact of tobacco cessation on buccal mucosa cancer risk: A multi-centre case-control study in India.

Authors list: Gayathri B. Pullat, Bastian Bohrmann, Grace Sarah George, Shubham Dikshit, Arjun Singh, Pankaj Chaturvedi, Rajesh Dikshit, Sarah Lewington, Sharayu Mhatre

Table of Contents

I. Appendix Materials and Methods 1
Appendix A: Calculation of duration of tobacco cessation 1
Appendix B: Calculation of alcohol and dietary consumption 1
Appendix C: Prevalence of quitters and users of tobacco products in the study 2
II. Appendix Tables 3
Appendix Table 1: List of study centres 3
Appendix Table 2: The inclusion criteria for the study participants 3
Appendix Table 3: Different kinds of tobacco products asked for in the study questionnaire 5
Appendix Table 4: Prevalence of users and quitters among different kinds of tobacco products in BMC 14
Appendix Table 5 (A): Odds ratios (95% CIs) of smoking tobacco status and quitting duration with risk of BMC 17
Appendix Table 5 (B): Odds ratios (95% CIs) of chewing tobacco status and quitting duration with risk of BMC 17
Appendix Table 5 (C): Odds ratios (95% CIs) of chewing tobacco status (with and without areca nut) and quitting duration with risk of BMC 18
Appendix Table 6: Risk due to individual tobacco products on development of BMC 20
Appendix Table 7 (A): Odds ratios (95% CIs) of smoking tobacco status (among the current chewers) and quitting duration with risk of BMC 22
Appendix Table 7 (B): Odds ratios (95% CIs) of chewing tobacco status (among the current smokers) and quitting duration with risk of BMC 23
III. Appendix Figures 24
Appendix Figure 1: Zones of India used for matching cases and controls 24
References 25

I. Appendix Materials and Methods

Appendix A: Calculation of duration of tobacco cessation

The duration of cessation among former users was calculated for ‘any smoking tobacco products’, ‘any chewing tobacco product’ and, ‘any chewing tobacco products with or without areca nut’.

Duration of tobacco cessation (for any tobacco product) = Age at the time of interview – Age at which the usage of the specific product was stopped.

Duration of cessation categories could not be generated for the former users of individual tobacco products due to insufficient numbers in each of the stratum – ‘<5 years’, 5-<10 years and ‘≥10 years’

Appendix B: Calculation of alcohol and dietary consumption

Alcohol consumption:

Alcohol consumption was calculated and adjusted as grams consumed per day, which was calculated using the following equation –

Alcohol grams per day = Number of glasses per day x 187.5* x 0.789 x percentage alcohol [5]

Where,

187.5 = 187.5 ml is the volume of beverage in 1 glass of drink

0.789 = Density of alcohol in g/m^3

The volume in a glass is determined through the approximation that a bottle of 750 ml makes up for 4 glasses.

Fruit and vegetable intake:

Information on the fruit and vegetable intake was collected from a comprehensive food frequency questionnaire, taking into account the seasonality of the most commonly consumed foods in the country. The frequency of consumption of every food item was collected in either number of times consumed per day, week, month or years. The data was then made uniform to consumption per day. The total fruit and vegetable consumption were generated as the maximum frequency of consumption of any of the fruit or vegetable, respectively.

The total fruit and vegetable intake per day was then categorised into- <2 versus ≥2 portions per day.

For any fruit intake the following were considered - Banana, Orange, Sweet lime, Lemon, Apple, Mango, Melon, Watermelon, Pear, and, Grape.

For any vegetable intake the following were considered - Tomatoes, Lady finger, Eggplant, Pumpkin, Drumstick, Beans, Bitter gourd, gourd, Ridged gourd, Sponge gourd, Ivy gourd, Colocasia, Carrot, Radish, Beet root, Potatoes, Turnip, Cauliflower, Cabbage, Fenugreek leaves, Spinach, Cow pea leaves, Bottle gourd, Ginger, Turmeric, Garlic and, Onions.

Appendix C: Prevalence of quitters and users of tobacco products in the study

Prevalence (%) of total users for any individual tobacco product = number of users / no. of any type of chewing or smoking tobacco users x 100 (for cigarette: 1014 / 1357 x 100 = 74.72%)

Prevalence (%) of number of quitters among the users for any individual tobacco product = number of quitters / no. of users for individual tobacco products x 100 (for cigarette: 418 / 1,014 x 100 = 24.56%)

Prevalence (%) of users among the cases for any product = number of users / total no. of cases for smoking / chewing tobacco products x 100 (for cigarette: 539 / 775 x 100 = 69.55%)

Prevalence (%) of number of quitters among the users in cases for any individual tobacco product = number of quitters / no. of users among the cases for individual tobacco products x 100 (for cigarette: 217 / 539 x 100 = 40.24%)

Prevalence (%) of users among the controls for any product = number of users / total no. of controls for smoking / chewing tobacco products x 100 (for cigarette: 582 / 475 x 100 = 81.62%)

Prevalence (%) of number of quitters among the users in controls for any individual tobacco product = number of quitters / no. of users among the controls for individual tobacco products x 100 (for cigarette: 201/ 475 x 100 = 43.31%

II. Appendix Tables

Appendix Table 1: List of study centres

Serial No.	Study centres	Institute Ethical Committee (IEC) approval number	Location
1.	Tata Memorial Hospital (TMH), TMC	IEC/0918/3114/001	Mumbai, Maharashtra
2.	Advanced Centre for Treatment Research and Education in Cancer (ACTREC), TMC	IEC/0918/3114/001	Navi Mumbai, Maharashtra
3.	Nargis Dutt Memorial Cancer Hospital (NDMCH)	IEC NDMCH/2019/01	Barshi, Maharashtra
4.	Dr B Borooah Cancer Institute (BBCI), TMC	BBCI-TMC/Misc-119/3195 /2018	Guwahati, Assam
5.	Homi Bhabha Cancer Hospital (HBCH) and Mahamana Pandit Madan Mohan Malaviya Cancer Centre (MPMMCC), TMC	2018/EC/708	Varanasi, Uttar Pradesh

Appendix Table 2: The inclusion criteria for the study participants

Serial No.	BMC	Visitors control
1.	All new histopathologically confirmed, primary sites of buccal mucosa of the following definition –<br>• Cheek mucosa ((C06·0)<br>• Retromolar trigones (C06·2),<br>• Bucco-alveolar sulci, and upper and lower vestibule of mouth (C06·1)<br>• Upper alveolus (C03.0)	Any visitor accompanying the patients to the hospitals who fulfils the following criteria:<br>• Friends<br>• Neighbours<br>• Second-degree relatives accompanying patients of any cancer site.<br>• First degree relatives accompanying patients of any cancer site except head and neck cancers

	• Lower alveolus (C03.1)	
2.	Male/female	Male/female
3.	Date of diagnosis less than or equal to 6 months from the date of enrolment.	
4.	Aged between 19-75 years	Aged between 19-75 years
5.	Have been a resident of India for at least a year.	Have been a resident of India for at least a year.

Appendix Table 3: Different kinds of tobacco products asked for in the study questionnaire

Sr. no.	Product type	Local name	Mode of use[1]	Product components/description	Pictures[#]
**1**	**Chewing tobacco product**				
**Products with areca nut**					
1.1	Betel quid with tobacco	*Paan*	Placed in the mouth and chewed.	Betel leaf containing tobacco, areca nut, slaked lime, catechu and, condiments wrapped into it.[2]	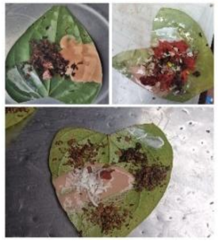 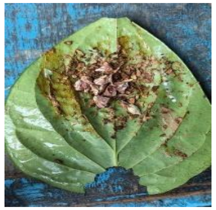
1.2	Mawa	*Mawa*	Held in the mouth and chewed	Contains tobacco flakes, areca nut shavings and slaked lime.[1]	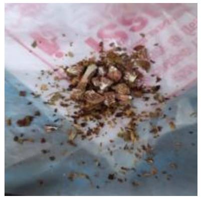 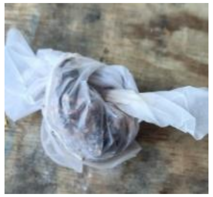
1.3	Gutkha	*Gutkha/Guthka*	Held in the mouth, sucked and chewed.	Available in two packets. Contains tobacco, areca nut, slaked lime, catechu, condiments.[2]	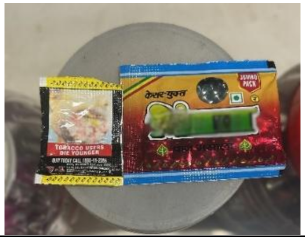

1.4	Dohra	*Dohra/Dhora*	Placed in the mouth, chewed and sucked.	A moist blend of tobacco, areca nut, slaked lime, catechu (*kattha*), peppermint and cardamom (*elaichi*).[2]	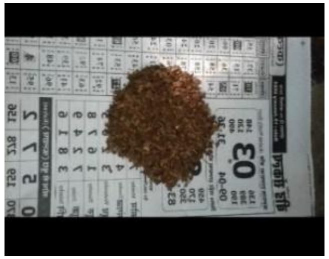
1.5	Kharra	*Kharra*	Held in the mouth, sucked and chewed.	Locally made. Contains tobacco, areca nut, slaked lime, catechu, and, condiments.[2]	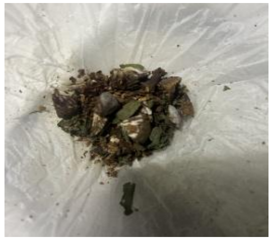
Products without areca nut					
1.6	Tobacco with lime	Khaini	Placed in the mouth and chewed.	Tobacco consumed with slaked lime.[2]	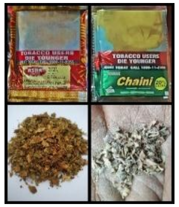

					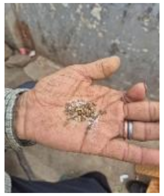 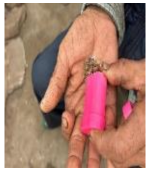
1.7	Pan masala: commercially in packets*	Pan masala	Placed in the mouth and chewed.	Pan Masala contains fennel seeds as the base ingredient as well as sugar coated seeds that may include sesame, fennel, and coriander seeds; mint leaves, cardamom, powdered lime, pure menthol, catechu, and other flavourings.	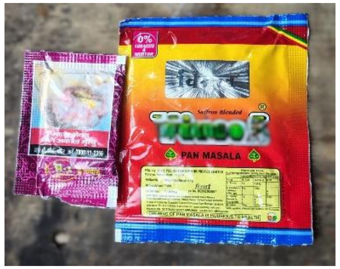 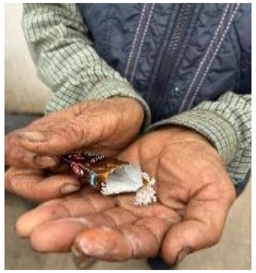

1.8	Dry Snuff	*Tapkeer/Bajar*[1]	Oral or nasal use	Powdered tobacco in dry form, available in plain or scented varieties.[1]	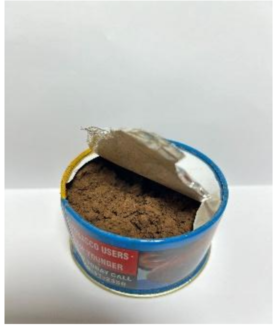
1.9	Zarda	Vizapatta[1]	Chewed alone or mixed with lime	Flavoured tobacco flakes are combined with aromatic spices, menthol, herbs, fragrances, saffron, raw kiwam, silver flakes, and sandalwood oil. When added saffron, it's called "Zafrani Zarda".[1]	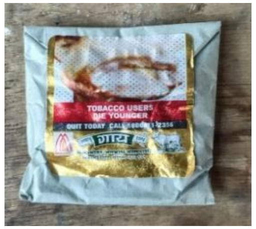 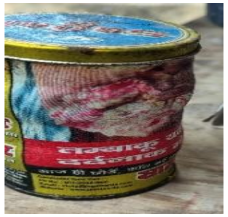

1.10	Kiwam	Kiwam	Placed in the mouth and chewed or used as an optional ingredient in betel quid.	Along with tobacco extract, also contains spices (saffron or cardamom or aniseed) and musk.[1]	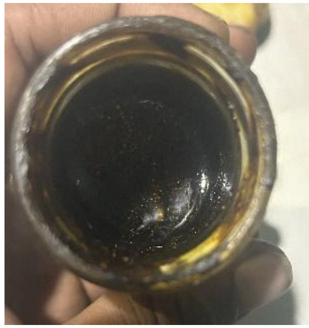 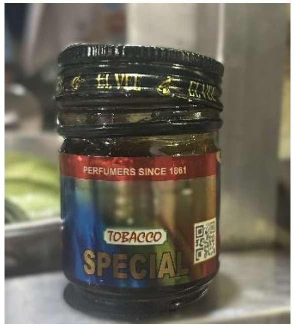
Tobacco for application					
1.11	Mishri	Mishri	Applied to the gums using a finger	Contains roasted and powdered tobacco.[1]	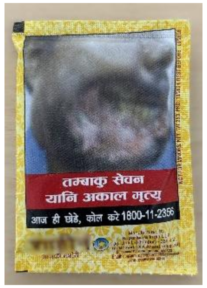

1.12	Tobacco containing tooth powder/toothpaste	Lal dantamanjan	Applied on teeth and gums as dentifrice.	Fine red powder that contains tobacco, herbs, and, flavourings. Additionally ginger, pepper, and, camphor may also be used.[1]	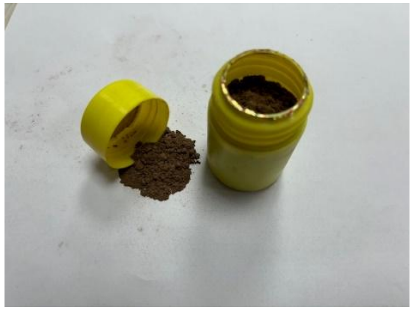
1.13	Gudakhu	Gudakhu	Applied on to the teeth and gums using the fingers. Some swallow the exact while others spit it out.	Tobacco and *sheera* (molasses), made into a paste[2]. May also contain lime and red soil.[1]	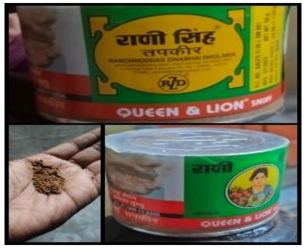

1.14	Gul	Gul	Applied on teeth and gums as dentifrice.	Pyrolysed tobacco powder[2] may also contain ash of tendu leaves.[1]	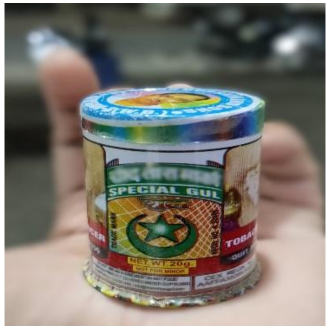
2	Smoking tobacco products				
2.1	Cigarette	Cigarette or *'sutta'*	Smoked	Thin roll of shredded or reconstituted tobacco, processed with hundreds of chemicals and rolled into a paper-wrapped cylinder.[3]	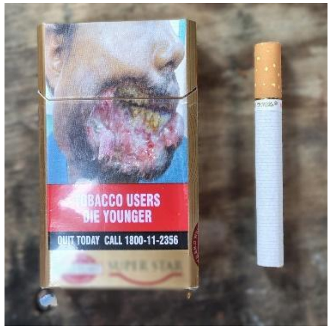
2.2	Bidi	Bidi or *Beedi*	Smoked	Thin, sun-dried, flaked tobacco hand wrapped in dried tendu leaf (*Diospyros sp.*) and tied with a string.[3]	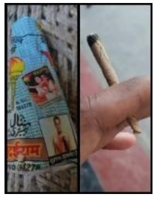 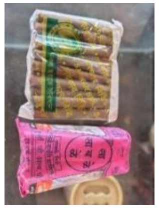 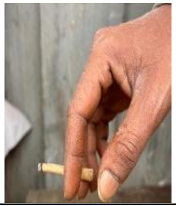

2.3	Hookah or waterpipe	Hookah	Inhaled	Operate by water filtration and indirect heat. Flavoured tobacco is burned in a smoking bowl covered with foil and coal. The smoke is cooled by filtration through a basin of water and consumed through a hose and mouthpiece.[3]	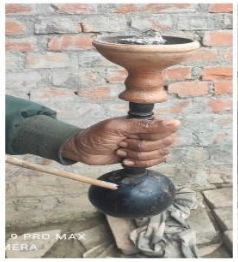 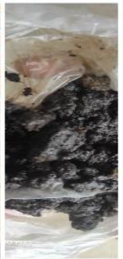
2.4	Chillum	Chillum/chilam	Inhaled	Chillum consists of a clay pipe 10cm-15cm long that is held vertically and is filled with tobacco.[4]	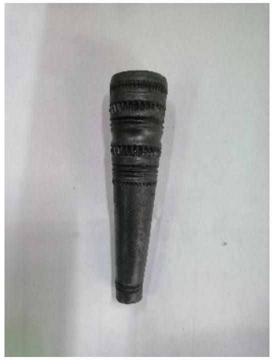
2.5	Cigar	Cigar	Smoked	Made up of a single type of air-cured or dried tobacco, tightly rolled, after being fermented in a multi-step process.[3]	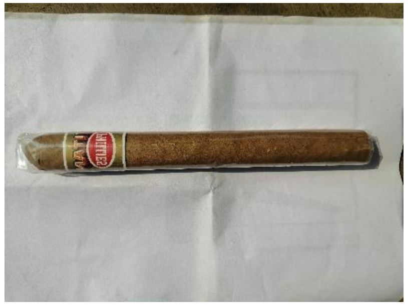

* The definitions for certain tobacco products were developed internally due to the observed geographical variations in their content.

# Pictures of the products were self-captured from different parts of India.

Appendix Table 4: Prevalence of users and quitters among different kinds of tobacco products in BMC

	Study population		Cases		Controls	
	Number of ever users (%) (N= 4,622)	Number of quitters among the users (%)	Number of ever users (%) (N_1=2,320)	Number of quitters among the users (%)	Number of ever users (%) (N_2=2,302)	Number of quitters among the users (%)
1. Chewing tobacco						
Any type of chewing product*	2,881 (62.33)	792 (27.49)	2,089 (90.04)	613 (29.34)	792 (34.40)	179 (22.60)
Tobacco with lime	1,769 (61.40)	435 (24.59)	1272 (60.89)	335 (26.33)	497 (62.75)	100 (20.12)
Gutkha	1,113 (38.63)	437 (39.26)	929 (44.47)	381 (41.01)	184 (23.23)	56 (30.41)
Betel quid with tobacco	922 (32.00)	310 (33.62)	692 (33.13)	243 (35.11)	230 (29.04)	67 (29.13)
Mawa	170 (5.90)	64 (37.65)	125 (5.98)	52 (41.60)	45 (5.68)	12 (26.66)
Mishri	127 (4.41)	55 (43.31)	100 (4.79)	41 (41.00)	27 (3.41)	14 (51.85)
Zarda	114 (3.96)	41 (35.96)	98 (4.69)	38 (38.91)	16 (2.02)	3 (18.25)
Gul	97 (3.37)	29 (29.90)	79 (3.78)	26 (32.23)	18 (2.27)	3 (16.67)
Panmasala: commercially in packets	77 (2.67)	32 (41.56)	52 (2.49)	21 (40.38)	25 (3.16)	11 (44.00)
Dry Snuff	19 (0.66)	6 (31.58)	14 (0.67)	3 (21.42)	5 (0.63)	3 (60.00)

Dohra	12 (0.42)	5 (41.67)	12 (0.57)	5 (41.67)	-	-
Kharra	13 (0.45)	7 (53.85)	12 (0.57)	7 (58.33)	1 (0.13)	-
Gudakhu	12 (0.42)	5 (41.67)	8 (0.38)	3 (37.5)	4 (0.51)	2 (50.00)
Toothpowder/ Toothpaste with tobacco	13 (0.45)	5 (38.46)	11 (0.53)	5 (45.45)	2 (0.25)	-
Khiwam	6 (0.21)	3 (50.00)	5 (0.24)	2 (40.00)	1 (0.13)	1 (100.00)
2. Smoking tobacco						
Any type of smoking product*	1,357 (29.36)	530 (39.06)	775 (33.41)	292 (37.68)	582 (25.28)	238 (40.89)
Cigarette	1,014 (74.72)	418 (24.56)	539 (69.55)	217 (40.24)	475 (81.62)	201 (42.31)
Bidi	543 (40.01)	193 (19.71)	379 (48.90)	130 (34.30)	164 (28.18)	63 (38.04)
Chillum	24 (1.77)	9 (16.66)	23 (2.97)	8 (34.78)	1 (0.17)	1 (100)
Hukka	12 (0.88)	5 (41.67)	6 (0.77)	2 (33.33)	6 (1.03)	3 (50.0)
Cigar	2 (0.15)	1 (50.00)	2 (0.26)	1 (50.00)	-	-
Dhuranti	2 (0.15)	1 (50.00)	1 (0.13)	-	1 (0.04)	1 (100.00)

Apart from the above, some tobacco products which went unreported but were included in our data collection are - Tuibur, Cheroot, Mainpuri, Handrolled tobacco, Chutta, Reverse chutta and Reverse dhuranti.
N = Total number of study participants
N1 = Total number of cases in the study
N2 = Total number of controls in the study
* These numbers do not represent the exclusive count of users and quitters for a specific tobacco product.
[#] Calculation of the prevalance has been explained in the appendix - Extended methods, pg. 9
Abbreviations used: BMC; Buccal mucosa cancer
Product definition:
Tobacco with Lime: Tobacco consumed with slaked lime.
Gutkha: Available in packets. Contains tobacco, areca nut, slaked lime, catechu, condiments.
Betel quid with tobacco: Betel leaf containing tobacco, areca nut, slaked lime, catechu and, condiments wrapped into it.
Mawa: Contain tobacco flakes, areca nut shavings and slaked lime.
Mishri: Contains roasted and powdered tobacco. Applied on to the teeth and gum.
Zarda: Flavoured tobacco flakes are combined with aromatic spices, menthol, herbs, fragrances, saffron, raw kiwam, silver flakes, and sandalwood oil.
Gul: Pyrolysed tobacco powder may also contain ash of tendu leaves, applied on to the teeth and gum.
Panmasala(commercially in packets): Pan Masala contains fennel seeds as the base ingredient as well as sugar coated seeds that may include sesame, fennel, and coriander seeds; mint leaves, cardamom, powdered lime, pure menthol, catechu, and other flavourings.
Dry Snuff: Powdered tobacco in dry form, available in plain or scented varieties.
Dohra: A moist blend of tobacco, areca nut, slaked lime, catechu (kattha), peppermint and cardamom (elaichi).
Kharra: Locally made. Contains tobacco, areca nut, slaked lime, catechu, and, condiments.
Gudakhu: Tobacco and sheera (molasses), made into a paste. May also contain lime and red soil, applied on to the teeth and gum.
Toothpowder/Toothpaste with tobacco: Wet mixture or fine red powder that contains tobacco, herbs, and, flavourings, applied on to the teeth and gum.
Khiwam: Along with tobacco extract, also contains spices (saffron or cardamom or aniseed) and musk.
Cigarette: Thin roll of shredded or reconstituted tobacco, processed with hundreds of chemicals and rolled into a paper-wrapped cylinder.
Bidi: Thin, sun-dried, flaked tobacco hand wrapped in dried tendu leaf (Diospyros sp.) and tied with a string.
Chillum: Chillum consists of a clay pipe 10cm-15cm long that is held vertically.

Hukka: A hookah is a water pipe used to smoke flavoured tobacco.
Cigar: Made up of a single type of air-cured or dried tobacco, tightly rolled, after being fermented in a multi-step process.
Dhuranti: Similar to cigar, made by rolling tobacco leave in the another plant.

Appendix Table 5 (A): Odds ratios (95% CIs) of smoking tobacco status and quitting duration with risk of BMC

Variables	Number of cases	OR (95% CI)
Current smokers	482	1.00 (0.86-1.17)
Ceased smoking <5 years ago	119	1.32 (0.94-1.86)
Ceased smoking 5-10 years ago	57	0.83 (0.52-1.34)
Ceased smoking ≥10 years ago	116	0.39 (0.28-0.54)

* Adjusted for Age (continuous), Gender (Categorical), Education (categorical), Maximum duration of chewing tobacco (years, continuous), amount of alcohol consumed (grams/day, continuous), fruit consumption frequency per day (categorical), vegetable consumption frequency per day (categorical), BMI (categorical)
95% CIs are determined by the variance of the log risk (see *Methods*)
Abbreviations:
BMC: Buccal Mucosa Cancer
OR: Odds Ratio
CI: Confidence Interval

Appendix Table 5 (B): Odds ratios (95% CIs) of chewing tobacco status and quitting duration with risk of BMC

Variables	Number of cases	OR (95% CI)
Current chewers	1475	1.00 (0.90-1.11)

Ceased chewing <5 years ago	389	2.32 (1.79-3.01)
Ceased chewing 5-10 years ago	112	1.33 (0.90-1.98)
Ceased chewing ≥10 years ago	112	0.58 (0.43-0.81)

* Adjusted for Age (continuous), Gender (Categorical), Education (categorical), Maximum duration of smoking tobacco (years, continuous), amount of alcohol consumed (grams/day, continuous), fruit consumption frequency per day (categorical), vegetable consumption frequency per day (categorical), BMI (categorical)
95% CIs are determined by the variance of the log risk (see *Methods*)
Abbreviations:
BMC: Buccal Mucosa Cancer
OR: Odds Ratio
CI: Confidence Interval

Appendix Table 5 (C): Odds ratios (95% CIs) of chewing tobacco status (with and without areca nut) and quitting duration with risk of BMC

Variables		Number of cases	OR (95% CI)
Current users	Without areca nut	490	1.00 (0.84-1.19)
	With areca nut	383	1.86 (1.53-2.26)
Ceased chewing <5 years ago	Without areca nut	111	2.40 (1.60-3.62)
	With areca nut	168	5.41 (3.56-8.21)
Ceased chewing 5-10 years ago	Without areca nut	38	1.14 (0.63-2.06)

	With areca nut	49	4.65 (2.34-9.24)
Ceased chewing ≥10 years ago	Without areca nut	43	0.70 (0.43-1.14)
	With areca nut	48	1.66 (0.96-2.77)

* Adjusted for Age (continuous), Gender (Categorical), Education (categorical), Maximum duration of smoking tobacco (years, continuous), Maximum duration of chewing tobacco (years, continuous), amount of alcohol consumed (grams/day, continuous), fruit consumption frequency per day (categorical), vegetable consumption frequency per day (categorical), BMI (categorical)
95% CIs are determined by the variance of the log risk (see *Methods*)
Abbreviations:
BMC: Buccal Mucosa Cancer
OR: Odds Ratio
CI: Confidence Interval

Appendix Table 6: Risk due to individual tobacco products on development of BMC

Variables		Case/Control	OR* (95% CI)	p value
Smoking tobacco products				
Any Smoking Product #	Ever	775/582	Ref	
	Never	1545/1720	0.67 (0.57-0.78)	≤0.001
Cigarette	Ever	539/475	Ref	
	Never	1781/1827	0.81 (0.65-1.01)	0.056
Bidi	Ever	379/164	Ref	
	Never	1941/2138	0.44 (0.34-0.57)	≤0.001
Chewing tobacco products				
Any Chewing Product #	Ever	2089/792	Ref	
	Never	231/1510	0.05 (0.05-0.07)	≤0.001
Betel quid with tobacco	Ever	692/230	Ref	
	Never	1628/2071	0.53 (0.44-0.64)	≤0.001

Tobacco with lime	Ever	1272/497	Ref	
	Never	1048/1805	0.54 (0.46-0.64)	≤0.001
Gutkha	Ever	929/184	Ref	
	Never	1389/2118	0.13 (0.11-0.16)	≤0.001
Mawa	Ever	125/45	Ref	
	Never	2192/2257	0.48 (0.33-0.71)	≤0.001
Mishri	Ever	100/27	Ref	
	Never	2214/2275	0.77 (0.46-1.28)	0.314

* Adjusted for Age (continuous), Gender (categorical), Education (categorical) , BMI (continuous), frequency of vegetable intake per day (continuous), frequency of fruit intake per day (continuous), amount of alcohol consumed (grams/day, continuous), Maximum duration of tobacco chewing (continuous), Maximum duration of tobacco smoking (continuous)
# Adjusted only for Maximum duration of tobacco chewing (continuous) for the smoking analysis, and only Maximum duration of tobacco smoking (continuous) for the chewing analysis

Ever users are those individuals who have consumed the tobacco product at least once a week for six months or more.
Abbreviations:
BMC: Buccal mucosa cancer
OR: Odds Ratio
CI: Confidence Interval

Appendix Table 7 (A): Odds ratios (95% CIs) of smoking tobacco status (among the current chewers) and quitting duration with risk of BMC

Variables	Number of cases	Number of controls	OR (95% CI)
Current smokers and chewers	335	97	1.00 (0.78-1.29)
Ceased smoking <5 years ago	35	23	0.38 (0.21-0.66)
Ceased smoking 5-10 years ago	27	13	0.55 (0.28-1.11)
Ceased smoking ≥10 years ago	57	43	0.36 (0.23-0.57)

* Adjusted for Age (continuous), Gender (Categorical), Education (categorical), amount of alcohol consumed (grams/day, continuous), fruit consumption frequency per day (categorical), vegetable consumption frequency per day (categorical), BMI (categorical)

95% CIs are determined by the variance of the log risk (see *Methods*)

Abbreviations:

BMC: Buccal Mucosa Cancer

OR: Odds Ratio

CI: Confidence Interval

Appendix Table 7 (B): Odds ratios (95% CIs) of chewing tobacco status (among the current smokers) and quitting duration with risk of BMC

Variables	Number of cases	Number of controls	OR (95% CI)
Current smokers and chewers	335	97	1.00 (0.79-1.27)
Ceased chewing <5 years ago	60	10	1.58 (0.78-3.20)
Ceased chewing 5-10 years ago	26	7	0.99 (0.41-2.42)
Ceased chewing ≥10 years ago	15	7	0.70 (0.27-1.85)

* Adjusted for Age (continuous), Gender (Categorical), Education (categorical), amount of alcohol consumed (grams/day, continuous), fruit consumption frequency per day (categorical), vegetable consumption frequency per day (categorical), BMI (categorical)

95% CIs are determined by the variance of the log risk (see *Methods*)

Abbreviations:

BMC: Buccal Mucosa Cancer

OR: Odds Ratio

CI: Confidence Interval

III. Appendix Figures

Appendix Figure 1: Zones of India used for matching cases and controls

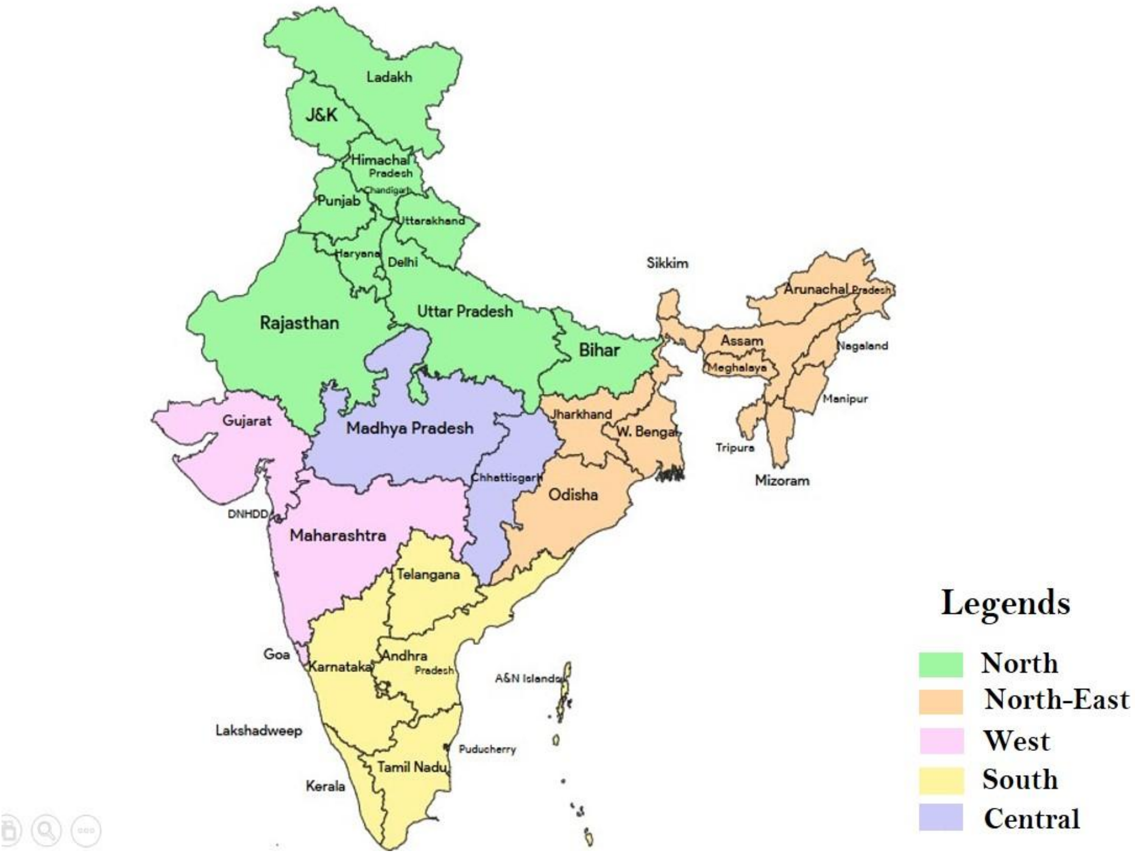

References

1 Smokeless tobacco (SLT) products | WHO FCTC. https://extranet.who.int/fctcapps/fctcapps/fctc/kh/slt/news/smokeless-tobacco-slt-products (accessed Oct 30, 2024).

2 Monograph on ‘Smokeless Tobacco and Public Health in India’ | Ministry of Health and Family Welfare | GOI. https://www.mohfw.gov.in/?q=basicpage/monograph-smokeless-tobacco-and-public-health-india (accessed Jan 3, 2025).

3 Mackay JL, Eriksen MP. The tobacco atlas. World Health Organization, 2002 https://iris.who.int/handle/10665/42580 (accessed Jan 3, 2025).

4 Singh S, Soumya M, Saini A, Mittal V, Singh UV, Singh V. Breath carbon monoxide levels in different forms of smoking. *Indian J Chest Dis Allied Sci* 2011; **53**: 25–8.

5 Alcohol Drinks & Grams of Alcohol - NutritionHeart.com. https://www.nutritionheart.com/alcohol-drinks-grams-of-alcohol/ (accessed April 8, 2025).
